# Diverse antidepressants increase CDP-diacylglycerol production and phosphatidylinositide resynthesis in depression-relevant regions of the rat brain

**DOI:** 10.1186/1471-2202-9-12

**Published:** 2008-01-24

**Authors:** Kimberly R Tyeryar, Habiba OU Vongtau, Ashiwel S Undieh

**Affiliations:** 1 Department of Pharmaceutical Sciences, Jefferson School of Pharmacy, Thomas Jefferson University, Philadelphia, PA 19107, USA; 2 Laboratory of Integrative Neuropharmacology, University of Maryland School of Pharmacy, Baltimore, MD 21201, USA

## Abstract

**Background:**

Major depression is a serious mood disorder affecting millions of adults and children worldwide. While the etiopathology of depression remains obscure, antidepressant medications increase synaptic levels of monoamine neurotransmitters in brain regions associated with the disease. Monoamine transmitters activate multiple signaling cascades some of which have been investigated as potential mediators of depression or antidepressant drug action. However, the diacylglycerol arm of phosphoinositide signaling cascades has not been systematically investigated, even though downstream targets of this cascade have been implicated in depression. With the ultimate goal of uncovering the primary postsynaptic actions that may initiate cellular antidepressive signaling, we have examined the antidepressant-induced production of CDP-diacylglycerol which is both a product of diacylglycerol phosphorylation and a precursor for the synthesis of physiologically critical glycerophospholipids such as the phosphatidylinositides. For this, drug effects on [^3^H]cytidine-labeled CDP-diacylglycerol and [^3^H]inositol-labeled phosphatidylinositides were measured in response to the tricyclics desipramine and imipramine, the selective serotonin reuptake inhibitors fluoxetine and paroxetine, the atypical antidepressants maprotiline and nomifensine, and several monoamine oxidase inhibitors.

**Results:**

Multiple compounds from each antidepressant category significantly stimulated [^3^H]CDP-diacylglycerol accumulation in cerebrocortical, hippocampal, and striatal tissues, and also enhanced the resynthesis of inositol phospholipids. Conversely, various antipsychotics, anxiolytics, and non-antidepressant psychotropic agents failed to significantly induce CDP-diacylglycerol or phosphoinositide synthesis. Drug-induced CDP-diacylglycerol accumulation was independent of lithium and only partially dependent on phosphoinositide hydrolysis, thus indicating that antidepressants can mobilize CDP-diacylglycerol from additional pools lying outside of the inositol cycle. Further, unlike direct serotonergic, muscarinic, or α-adrenergic agonists that elicited comparable or lower effects on CDP-diacylglycerol versus inositol phosphates, the antidepressants dose-dependently induced significantly greater accumulations of CDP-diacylglycerol.

**Conclusion:**

Chemically divergent antidepressant agents commonly and significantly enhanced the accumulation of CDP-diacylglycerol. The latter is not only a derived product of phosphoinositide hydrolysis but is also a crucial intermediate in the biosynthesis of several signaling substrates. Hence, altered CDP-diacylglycerol signaling might be implicated in the pathophysiology of depression or the mechanism of action of diverse antidepressant medications.

## Background

Major depression is an increasingly prevalent mood disorder that afflicts millions of people worldwide [1-3]. While a number of medications is available for managing the disease symptoms, the mechanism of action of current antidepressants is not well understood [4,5]. It is known, however, that antidepressant medications generally increase the synaptic levels of the monoamine neurotransmitters serotonin, norepinephrine, and/or dopamine in discrete brain regions [6,7]. The monoamines are then thought to activate their cognate postsynaptic receptors and modulate the activities of downstream signaling cascades to produce the antidepressive effect [5,8-10].

Monoamine receptors coupled to diverse signaling cascades, including those mediated through adenylyl cyclase, phospholipases, and MAP Kinases [11-14]. Aspects of each signaling system have been investigated as potential downstream targets of antidepressive mechanisms with multiple and sometimes divergent findings [8,15,16]. As examples, acute or chronic treatment with various antidepressant compounds can lead to changes in basal or drug-induced activities of brain adenylyl cyclase [17-20], phospholipase A2 [21], CREB [22,23], phosphoinositide-specific phospholipase C (PLC) [15,24,25], inositol phosphates (IPs) [26-29], phosphatidylinositides (PI) [29,30], protein kinase C (PKC) [31-33], extracellular signal regulated kinase [16,34], ion channels [35,36], neurotrophins [37-39], and various neuropeptides [40-42]. Antidepressants can also enhance neurogenesis [43-46], modulate neuronal excitability [47-49], and alter the gene expression of various signaling components including neurotransmitter transporters, receptors, transducers, and effectors [50-53]. While these observations suggest that changes in postsynaptic signaling cascades may constitute an integral component in the mechanisms that underlie depression or its treatment with antidepressant medications, no signaling cascade has been identified that adequately explains the behavioral and clinical data.

The PI-related observations in particular have been corroborated by clinical studies showing that depressed persons may have reduced cortical levels of the PI precursor *myo*-inositol [54,55]. Moreover, oral ingestion of bolus doses of *myo*-inositol could elicit antidepressive responses in rodents [56,57], and enhance the recovery of clinically depressed patients [58]. Consistent with these findings, chronic administration of antidepressant agents has been associated with increased levels of the PIs in human platelets [29,30]. These observations suggest that alterations in the PI signaling pathway may be implicated in the pathophysiology of depression and/or the mode of action of antidepressant agents [5,25,59,60].

Several past studies examined IP signaling, but not the status of diacylglycerol (DG) production or signaling, as a potential target of disease pathology or pharmacological treatment with antidepressants [31-33]. Diacylglycerol signaling is important because this lipid is the endogenous regulator of PKC activity, and the latter is one of the indices shown to be altered in depressed persons [31-33,61]. Moreover, PLC-stimulating receptors show differences in their capacity to generate diacylglycerol (relative to IP) from phospholipid hydrolysis [62,63]. Therefore, to the extent that PI signaling or PKC activity may be involved in antidepressant drug action, it should be important to clarify the specific effects of the agents on diacylglycerol production as a potential basis for their therapeutic efficacy. Following our previously reported preliminary observations [64], we have now examined antidepressant drug effects on cellular diacylglycerol production and metabolism, including the resynthesis of the PI substrates. The results show that antidepressants belonging to diverse chemical and pharmacological classes acutely increase the formation of CDP-diacylglycerol (CDP-DG), a metabolic derivative of diacylglycerol, and that this effect does translate to enhanced resynthesis of the PIs. The latter are physiologically critical not only as substrates for PLC signaling but also as mediators in the phosphatidylinositol-3-kinase (PI3K)/Akt signaling cascades. It is conceivable, therefore, that an acute molecular action of antidepressant agents that conserves or supplements cellular PIs could ultimately contribute to the therapeutic mechanism of these medications in depression.

## Results

### Chemically diverse antidepressant agents increase CDP-diacylglycerol production

**Figure 1:**
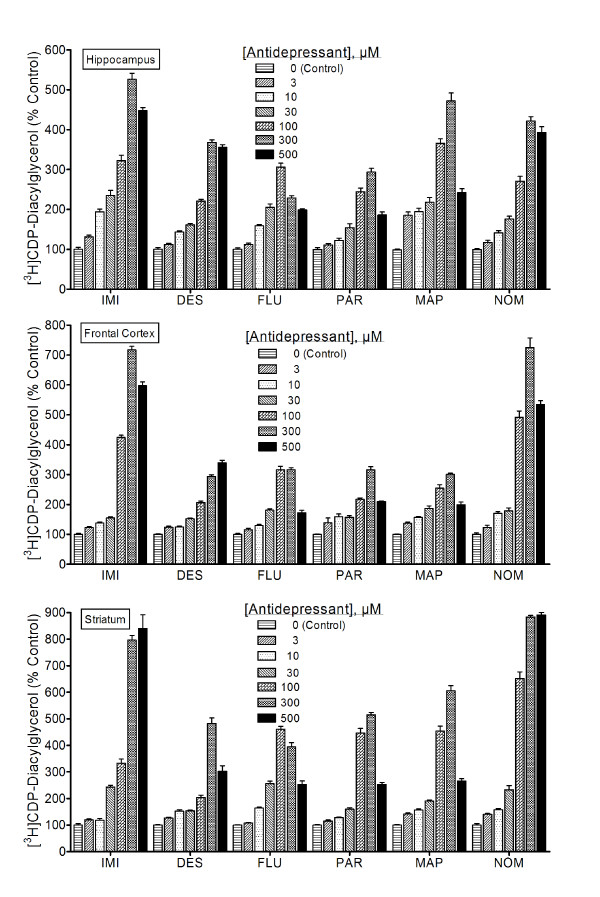
**Effects of classic antidepressants on [^3^H]CDP-diacylglycerol accumulation**. Tissue slices prepared from indicated brain regions were prelabeled with [^3^H]cytidine and incubated with various concentrations of either imipramine (IMI), desipramine (DES), fluoxetine (FLU), paroxetine (PAR), maprotiline (MAP), or nomifensine (NOM). After 90 min, tissue contents of [^3^H]CDP-diacylglycerol were assayed. Each bar is the mean ± SEM (N = 9). Each drug stimulated significant concentration-dependent accumulations of CDP-diacylglycerol (ANOVA, p < 0.001 for each drug). Based on *posthoc *Dunnett tests, all agents induced statistically significant CDP-diacylglycerol responses at the 3 or 10 μM concentrations, except for paroxetine in the hippocampus and imipramine in the striatum where the drug effects were not significant until the 30 μM and higher concentrations.

Diacylglycerol released from phospholipid breakdown is normally rapidly phosphorylated to produce phosphatidic acid. In the presence of [^3^H]cytidine-labeled CTP, however, the phosphatidic acid is converted to radiolabeled CDP-DG, which can be extracted and separated away from other labeled metabolites and subsequently quantified. In the present study, rat brain cerebrocortical, hippocampal, and striatal slices prelabeled with [^3^H]cytidine were incubated with various concentrations of selected antidepressant agents in the presence of LiCl, and the yield of CDP-diacylglycerol analyzed. Data for each drug were separately analyzed before they were normalized and collated together for graphical presentation as shown. The classical antidepressants imipramine and desipramine, the selective serotonin reuptake inhibitors fluoxetine and paroxetine, and the atypical agents maprotiline and nomifensine, each significantly and dose-dependently enhanced the accumulation of [^3^H]CDP-DG in rat hippocampal, prefrontal cortical, and striatal slices (Figure 1). While concentrations ranging from 0.1 to 1000 μM were tested, only those concentrations lying between the minimal that gave statistically significant effects for any agent (1–3 μM) and the maximally effective concentrations (100 – 500 μM) are shown. Statistically significant effects were obtained at concentrations as low as 3–10 μM in the hippocampus or prefrontal cortex, while maximal effects were achieved at the 100 μM concentration of fluoxetine or 300 μM concentrations of most other agents. For all agents, test concentrations greater than 300 μM resulted in CDP-DG effects that were either statistically similar to, or significantly lower than, the effects observed at 100 μM for fluoxetine or 300 μM for the other agents. This reduction in response with increasing concentration after attaining maximal responses was more apparent with the SSRIs, fluoxetine and paroxetine, than with the tricyclic agents.

Among the brain regions, the hippocampus appeared to be more sensitive (greater response magnitudes at lower concentrations), whereas the striatum gave slightly more robust (maximally attained) effects. The drug responses were statistically dose-dependent for all effective agents in each tissue, but there were noticeable differences in potency or efficacy among the compounds as shown in the data. Thus, diverse antidepressant agents can acutely induce CDP-DG synthesis in depression-relevant regions of the rat brain.

### Antidepressant-induced CDP-diacylglycerol formation translates into increased PI synthesis

**Figure 2:**
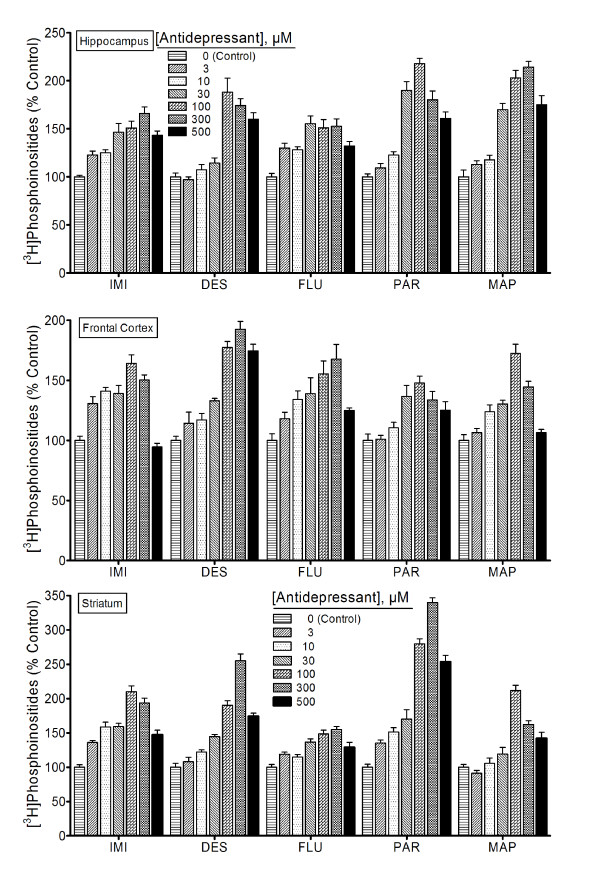
**Effects of various classic antidepressants on [^3^H]phosphatidylinositide synthesis**. Tissue slices prepared from indicated brain regions and prelabeled with [^3^H]inositol were incubated with various concentrations of either imipramine (IMI), desipramine (DES), fluoxetine (FLU), paroxetine (PAR), or maprotiline (MAP). After 90 min, [^3^H]inositol phospholipids were extracted and assayed as a total pool of extractable phosphatidylinositides. Each bar is the mean ± SEM (N = 9). Each drug stimulated significant and concentration-dependent increases in [^3^H]inositol phospholipid synthesis (ANOVA, p < 0.001 for each drug). From the subsequent *posthoc *Dunnett tests, all agents induced statistically significant CDP-diacylglycerol responses at the 3 or 10 μM and higher concentrations.

To test if the antidepressant-enhanced CDP-DG translates into increased synthesis of the PIs, brain slice preparations were labeled with [^3^H] inositol and incubated in the presence of various antidepressant agents. Results of the subsequent uptake and conversion of [^3^H] inositol into inositol phospholipids are shown in Figure 2. Imipramine, desipramine, fluoxetine, paroxetine, and maprotiline each significantly increased [^3^H]inositol labeling of PIs in the tested brain regions (Figure 2). Thus, the increased mobilization or recapture of CDP-DG by the antidepressant agents translates into increased regeneration of PI signaling substrates.

### Effects of monoamine oxidase inhibiting agents CDP-diacylglycerol formation and PI synthesis

**Figure 3:**
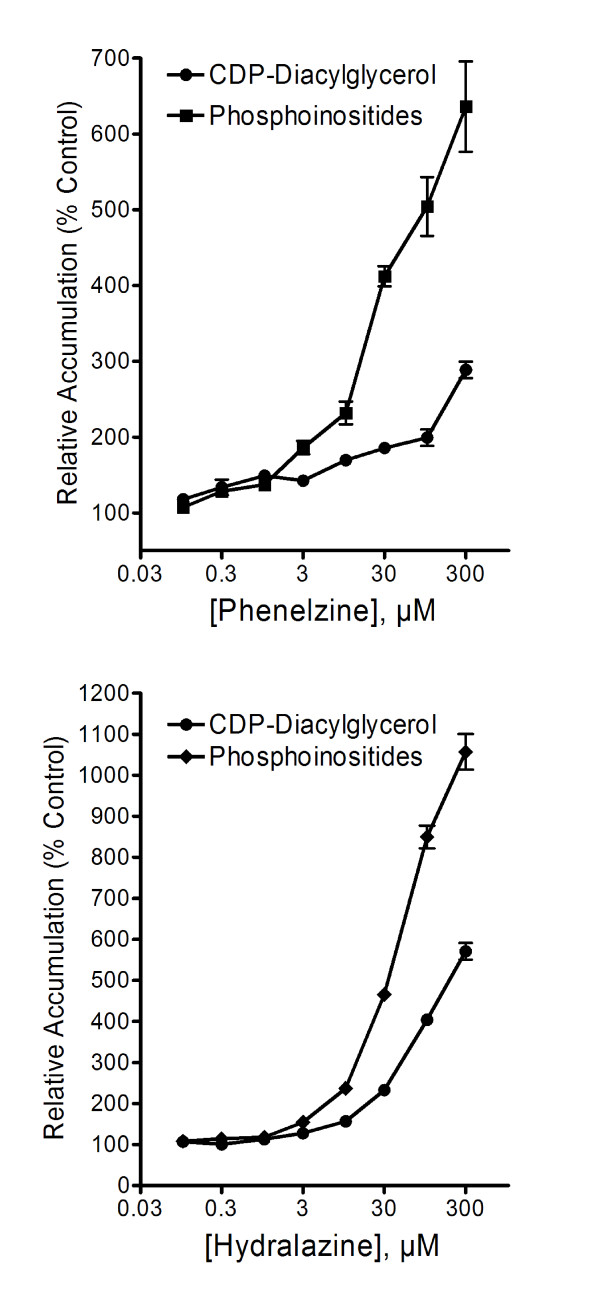
**Effects of the monoamine oxidase inhibitors on [^3^H]CDP-diacylglycerol accumulation and phosphatidylinositide synthesis**. Cortical slices were tested with indicated concentrations of phenelzine or hydralazine. CDP-diacylglycerol (CDP-DG) levels were analyzed as outlined under Figure 1, while phosphatidylinositide (PI) synthesis was measured as the total pool of extractable [^3^H]inositol-labeled phospholipids as in Figure 2. Each point is the mean ± SEM (N = 9). Each drug stimulated significant concentration-dependent accumulations of CDP-diacylglycerol (ANOVA, p < 0.001 for each drug) and phosphatidylinositide synthesis (ANOVA, p < 0.001 for each drug). Note that if individually compared to the corresponding control measure, each of the tested concentrations from 0.1 – 300 μM gave statistically significant effects on CDP-DG and PIs. Note also the greater relative accumulation of PIs compared to the accumulation of CDP-DG.

Similar to the effects exhibited by the classical antidepressant agents, the monoamine oxidase inhibitors (MAOIs), phenelzine and hydralazine, produced robust effects on CDP-DG accumulation in frontal cortex slices (Figure 3), while tranylcypromine had statistically significant but rather modest effects (data not shown). While the effects of phenelzine achieved significance at 1 μM (Dunnett's, p < 0.01), those of hydralazine became significant at the 10 μM and higher concentrations. Conversely, no significant effects were observed with several other tested MAOIs, namely, pargyline, selegiline, quinacrine, or clorgyline (Table 1).

**Table 1:** Psychotropic agents lacking effects on CDP-diacylglycerol accumulation in rat cerebrocortical slices. Agents were tested at multiple concentrations ranging from 0.1–300 μM. Data from up to three separate runs were normalized and pooled for analysis by One-Way ANOVA. None of these compounds showed significant or concentration-related effects on CDP-diacylglycerol.

*Antipsychotics*	*MAOIs*	*Anticonvulsants/Anxiolytics/Others*	
Chlorpromazine	Hydroxylamine	Benztropine	Phenylephrine
Haloperidol	Selegiline	Phenytoin	Phenobarbital
Sulpiride	Pargyline	Diazepam	Chlordiazepoxide
Flupenthixol	Quinacrine	Nitrazepam	

MAOIs that were effective in inducing CDP-DG production also showed enhanced effects on PI resynthesis (Figure 3), whereas other MAOIs that were ineffective on CDP-DG were equally ineffective in increasing PI resynthesis (data not shown). The MAOI data (Figure 3) also depict the significantly greater relative accumulation of PIs compared to the accumulation of CDP-DG, implying the conversion of CDP-DG to PI may be a dynamic or cumulative process.

We also tested a range of other psychotropic compounds in order to estimate the extent to which the CDP-DG response may characterize compounds with antidepressive activity. Neither the antipsychotic agents sulpiride, chlorpromazine and haloperidol, nor various other psychotropic compounds induced any significant effects on CDP-DG production (Table 1).

### Antidepressant Agents Generally Enhance Inositol Phosphate Accumulation

**Figure 4:**
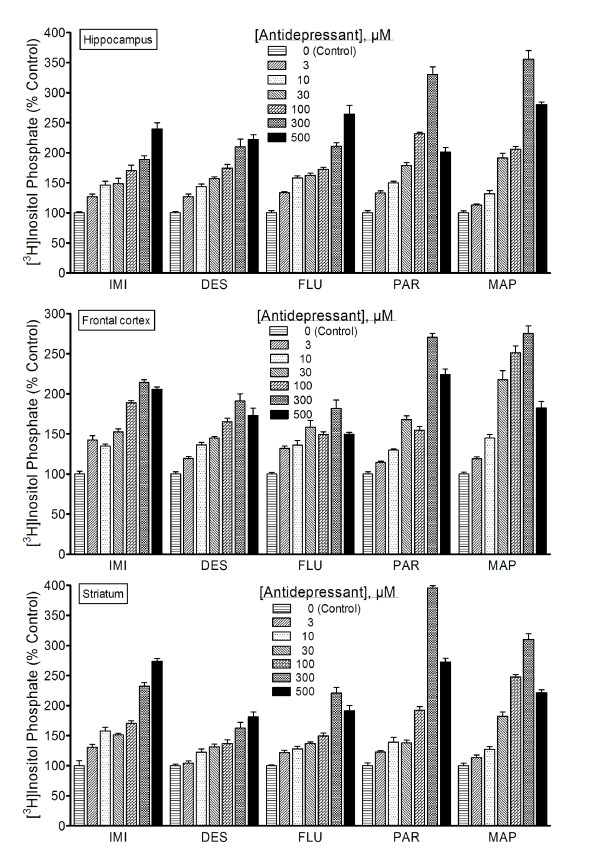
**Effects of diverse antidepressants on [^3^H]inositol phosphate accumulation**. Experiments were conducted as outlined in the legend to Figure 2, except that tissue contents of [^3^H]inositol phosphates were assayed by Dowex anion exchange chromatography as detailed in Methods. Each bar is the mean ± SEM (N = 9). Each antidepressant agent stimulated significant concentration-dependent accumulations of inositol phosphate (ANOVA, p < 0.001 for each drug).

To test if resynthesized PIs might contribute to enhanced IP accumulation, agents tested for effects on CDP-DG were also tested in a standard IP assay. Across a concentration range of 3–300 μM, imipramine, desipramine, fluoxetine, paroxetine, and maprotiline significantly and dose-dependently stimulated the accumulation of IPs in each brain region (Figure 4). Significant drug effects were generally evident at concentrations of 3–10 μM, while maximal effects were observed at 100–300 μM. With imipramine tested in the hippocampus and striatum as the only possible exceptions, test concentrations greater than 300 μM resulted in IP effects that were either statistically similar to, or significantly lower than, the effects observed at the corresponding 300 μM concentration. In general, drug concentrations greater than 300–500 μM were associated with IP levels that were significantly lower than effects at 100–300 μM concentrations, possibly reflecting toxicity from excessive stimulation.

### Antidepressant-induced CDP-diacylglycerol formation partially depends on PI hydrolysis

**Figure 5:**
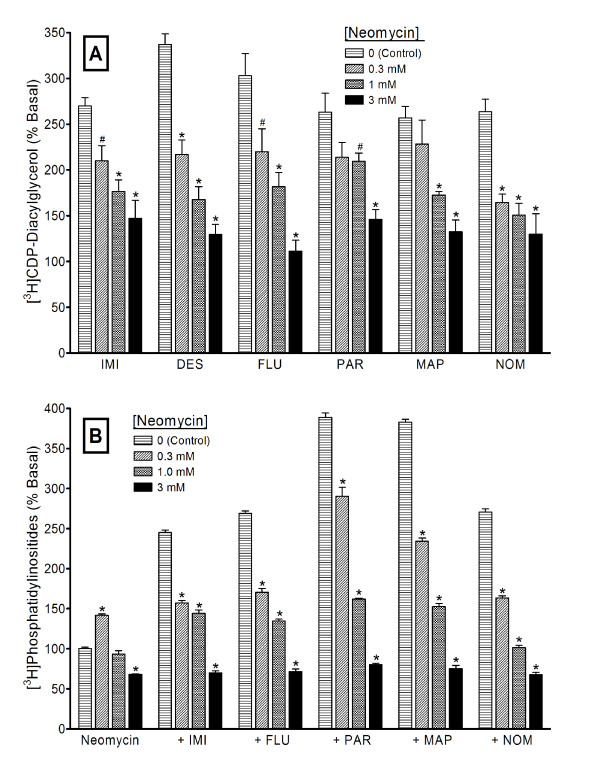
**Inhibition of antidepressant-induced [^3^H]CDP-diacylglycerol production and phosphoinositide synthesis by neomycin**. Slices of rat prefrontal cortex or hippocampus prepared from the same rats were pre-labeled in parallel with [^3^H]cytidine or [^3^H]inositol and incubated with indicated concentrations of neomycin, followed by addition of 100 μM fluoxetine (FLU) or 300 μM imipramine (IMI), desipramine (DES), paroxetine (PAR), maprotiline (MAP), or nomifensine (NOM). Accumulated [^3^H]CDP-diacylglycerol (**A**) or [^3^H]phosphoinositides (**B**) were measured after 90 min. While only the hippocampus data are shown for CDP-diacylglycerol and the cortical data for phosphatidylinositides, each analyte was assessed in each brain region with similar results. Each bar is the mean ± SEM (*n *= 6). Neomycin significantly and concentration-dependently inhibited drug-induced [^3^H]CDP-diacylglycerol production and [^3^H]inositol phospholipid synthesis (ANOVA, p < 0.01 for each drug). The effects of neomycin alone on CDP-diacylglycerol were not significant, whereas it exerted a slight but significant increase at the 0.3 mM concentration (Dunett's, p < 0.01) and decrease at the 3 mM concentration (Dunnett, p < 0.01) in [^3^H]phosphatidylinositide levels. #p < 0.05; *p < 0.05, compared to respective effects of antidepressant alone by Dunnett test.

**Figure 6:**
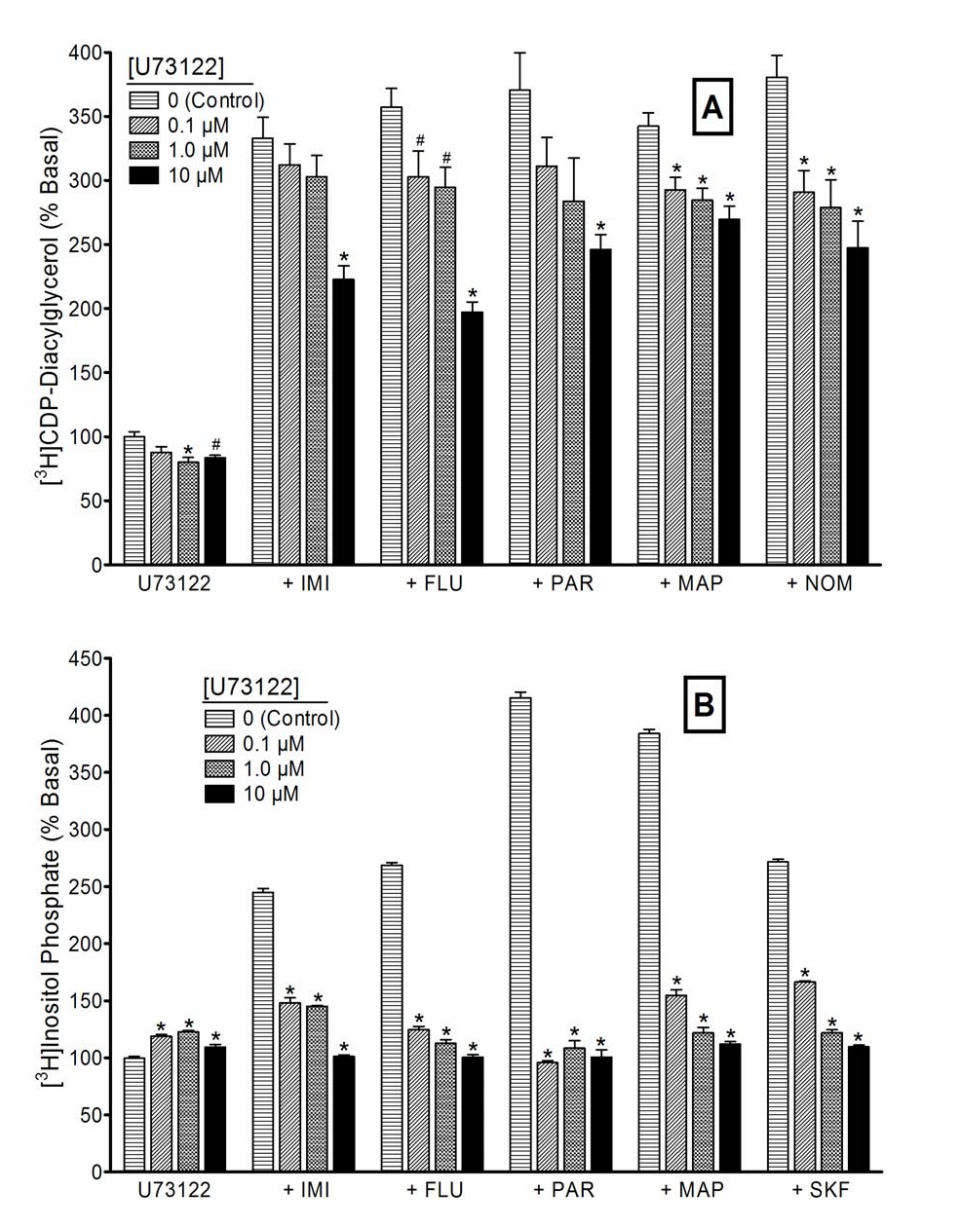
**Effects of the PLC inhibitor U73122 on antidepressant-mediated CDP-diacylglycerol production and inositol phosphate accumulation**. Cerebrocortical or hippocampal slices labeled with either [^3^H]inositol or [^3^H]cytidine were incubated in parallel with buffer alone or the indicated concentrations of U73122, followed by addition of 100 μM fluoxetine (FLU) or 300 μM imipramine (IMI), paroxetine (PAR), maprotiline (MAP), nomifensine (NOM), or SKF38393 (SKF) as indicated. Accumulated [^3^H]CDP-diacylglycerol (**A**) or [^3^H]inositol phosphates (**B**) were determined after 90 min. While only the hippocampus data are shown for inositol phosphates and the cortical data for CDP-diacylglycerol, each analyte was assessed in each brain region with similar results. Each bar is the mean ± SEM (*n *= 6). U73122 completely blocked [^3^H]inositol phosphate accumulation stimulated by either antidepressant agent (ANOVA, p < 0.001). Conversely, U73122 only partially reduced antidepressant drug-induced [^3^H]CDP-diacylglycerol production. The phospholipase C inhibitor by itself showed slight but significant effects on either analyte. *p < 0.01 compared to effects of antidepressant alone (Dunnett test).

Phosphoinositide hydrolysis is a major source, but not the only possible source, of diacylglycerol in the cell. To estimate the extent to which antidepressant-enhanced CDP-DG may derive from PI breakdown, we blocked PI hydrolysis and then measured the consequent effects on the ability of antidepressant agents to induce CDP-DG accumulation. First, we tested the effects of the general PI metabolism inhibitor, neomycin, against the maximally effective concentrations of the selected antidepressant agents. Neomycin concentration-dependently blocked the effects of imipramine, desipramine, fluoxetine, paroxetine, maprotiline, or nomifensine on CDP-DG production (Figure 5A), PI resynthesis (Figure 5B) or IP accumulation (data not shown) in hippocampal or prefrontal cortical brain slices. Increasing concentrations of neomycin produced complete blockade of both CDP-DG and PI responses. We next tested the effects of the selective PLC inhibitor, U73122, on the drug responses. As shown in Figure 6, U73122 by itself did not significantly alter basal CDP-DG production (Figure 6A) or IP accumulation (Figure 6B), although a slight increase in IP was consistently noted. At concentrations ranging from 0.1 to 10 μM, U73122 significantly reduced, but was unable to completely block, antidepressant drug effects on CDP-DG production. Conversely, the PLC inhibitor completely blocked IP stimulation by 100 μM fluoxetine or 300 μM concentrations of imipramine, paroxetine, maprotiline, or nomifensine in hippocampal or cortical slices. To validate the effects of U73122, we also tested the compound against the action of SKF38393, a D_1 _receptor agonist that is known to induce PI hydrolysis in these brain tissues [14,65]. SKF38393-induced IP accumulation was blocked by U73122 with similar efficacies to the inhibition of the antidepressant responses (Figure 6B). Moreover, U73123, an analog of U73122 that is ineffective in blocking PLC activity, was without effect on any of the CDP-DG or IP responses (data not shown). The effects of the SSRIs fluoxetine and paroxetine were more sensitive to inhibition by U73122 than the effects of the tricyclic agents.

### Lithium is not required for antidepressant drug effects on CDP-diacylglycerol

**Figure 7:**
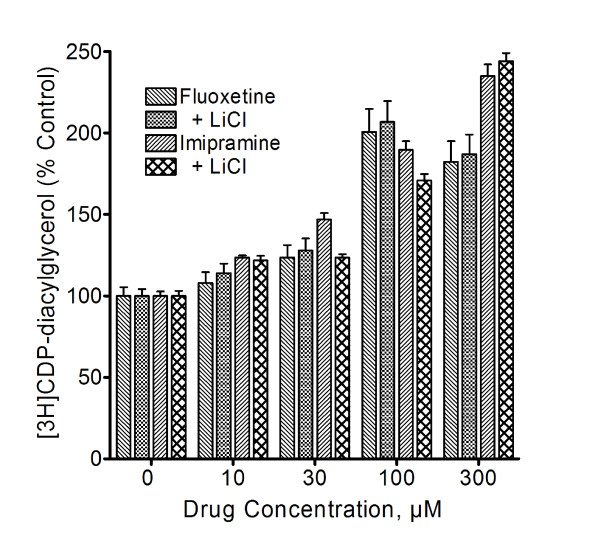
**Effects of LiCl on antidepressant-induced [^3^H]CDP-diacylglycerol production**. Slices of prefrontal cortical or hippocampal tissues were labeled with [^3^H]cytidine and incubated in the presence or absence of 5 mM LiCl. Indicated concentrations of fluoxetine or imipramine were added, and after 60 min accumulated [^3^H]CDP-diacylglycerol was measured. While the frontal cortex data are shown, similar observations were made in the hippocampus. Each bar is the mean ± SEM (*n *= 6). The presence of LiCl did not significantly alter the stimulatory effects of fluoxetine or imipramine on [^3^H]DCP- diacylglycerol accumulation (ANOVA, p > 0.05).

Our initial experiments were designed to compare antidepressant drug effects on the IP and diacylglycerol arms of the inositol cycle. Thus, it was necessary to include LiCl in all test incubations, seeing Li^+ ^is needed to block inositol monophosphatase and thereby enable the accumulation of released IPs to measurable levels. But, is the presence of Li^+ ^necessary to demonstrate antidepressant drug effects on CDP-DG? To answer this question we performed additional experiments in which selected antidepressant agents were tested for effects on CDP-DG in the absence or presence of 5 mM LiCl. As shown in Figure 7, LiCl did not significantly enhance or inhibit antidepressant drug-induced CDP-DG production, implying that the presence of Li^+ ^is not necessary to demonstrate the enhancing effects of antidepressant agents on CDP-DG production.

### Antidepressants elicit greater stimulation of CDP-diacylglycerol production than IP formation

**Figure 8:**
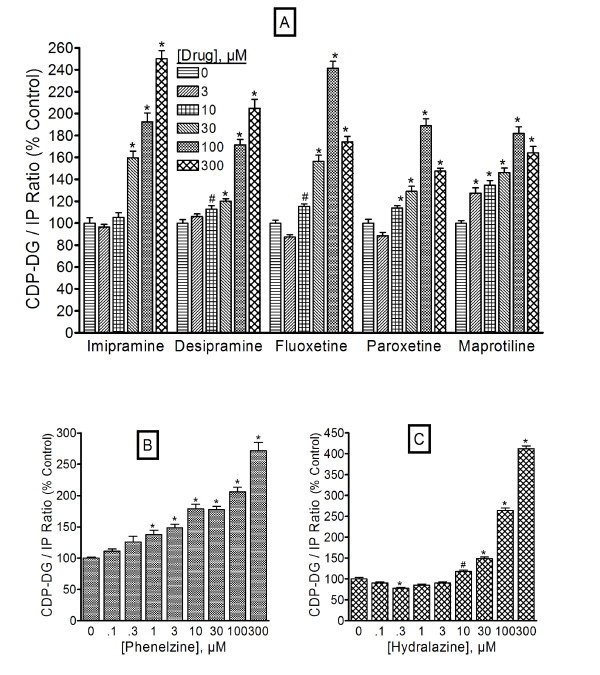
**Ratios of antidepressant-induced CDP-diacylglycerol versus inositol phosphates**. The data in Figures 1-4 were recalculated by dividing the CDP- diacylglycerol effects of each concentration of each antidepressant agent by the corresponding effects of the agent on inositol phosphate accumulation to yield the CDP- diacylglycerol/inositol phosphate (CDP-DG/IP) ratios shown. To facilitate merging of data from multiple experiments, these values were converted to percentages relative to the ratio values in the respective control samples and then averaged to give the mean ± SEM shown for the classic agents (**A**), phenelzine (**B**), and hydralazine (**C**). Data for the hippocampus are shown, but similar observations were made in the cortical tissues. Data for each agent were analyzed by One-way ANOVA followed by *posthoc *Dunnett tests. ^#^p < 0.05; *p < 0.01; compared to the respective control (zero drug concentration).

To determine if antidepressant agents exert differential effects on CDP-DG production compared to PI hydrolysis, we examined the ratios of CDP-DG production relative to the IPs (CDP-DG/IP ratio) in corresponding treatment conditions. The ratios were calculated from the data in Figures 1, 2, 3, 4 and the results are shown in Figure 8. With each antidepressant agent, the CDP-DG/IP ratios increased significantly with increasing drug concentrations. This was true for different classes of drugs, including the MAOIs phenelzine and hydralazine.

### Monoamine receptor agonists exert divergent effects on CDP-diacylglycerol

**Figure 9:**
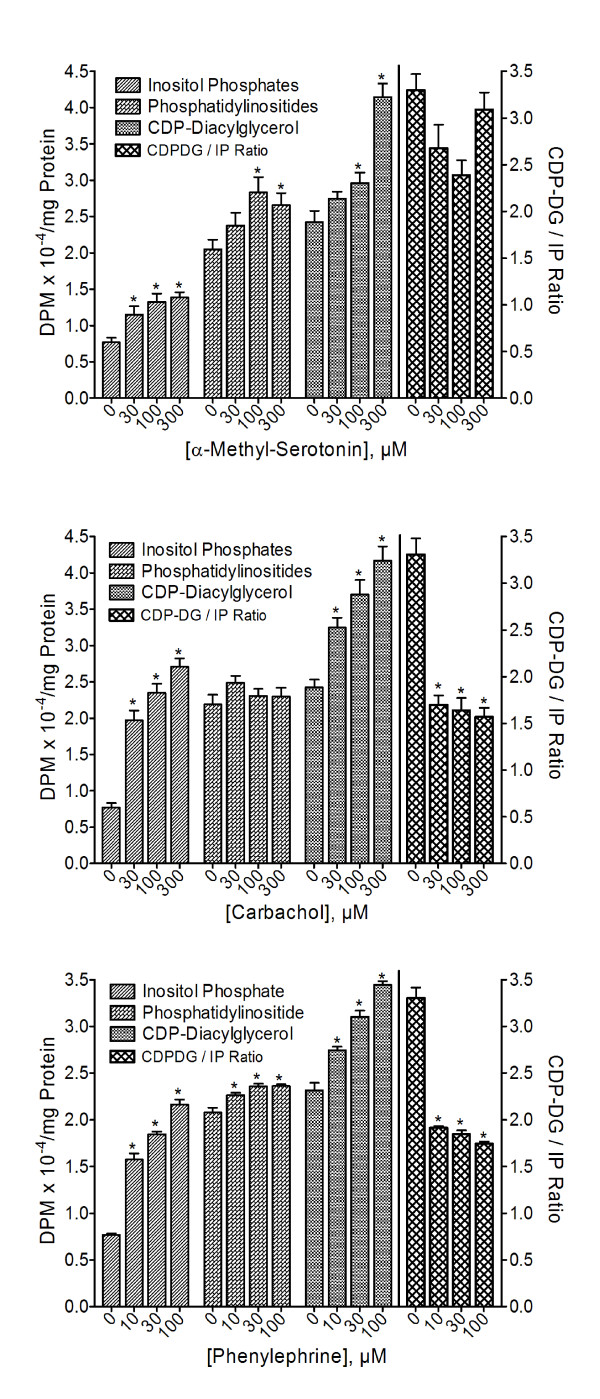
**Effects of α-methylserotonin, carbachol, and phenylephrine on CDP-diacylglycerol production, inositol phosphate accumulation and phosphatidylinositide synthesis**. Slices of prefrontal cortical or hippocampal tissues were pre-labeled with [^3^H]cytidine or [^3^H]inositol in parallel, and then incubated in the presence of 5 mM LiCl. Indicated concentrations of the agonists were added for 90 min, followed by assay of the levels of [^3^H]CDP- diacylglycerol, [^3^H]inositol phosphates, and [^3^H]phosphoinositides. Calculated ratios of CDP- diacylglycerol over inositol phosphates (CDP-DG/IP ratios) are shown in the rightmost panel relative to the scale on the right y-axis. Each bar is the mean ± SEM (*n *= 15 for α-methylserotonin, 12 for carbachol, 9 for phenylephrine). Data were separately analyzed by One-way ANOVA for each receptor agonist. *p < 0.01, Dunnett test compared to the respective control (zero drug concentration).

**Figure 10:**
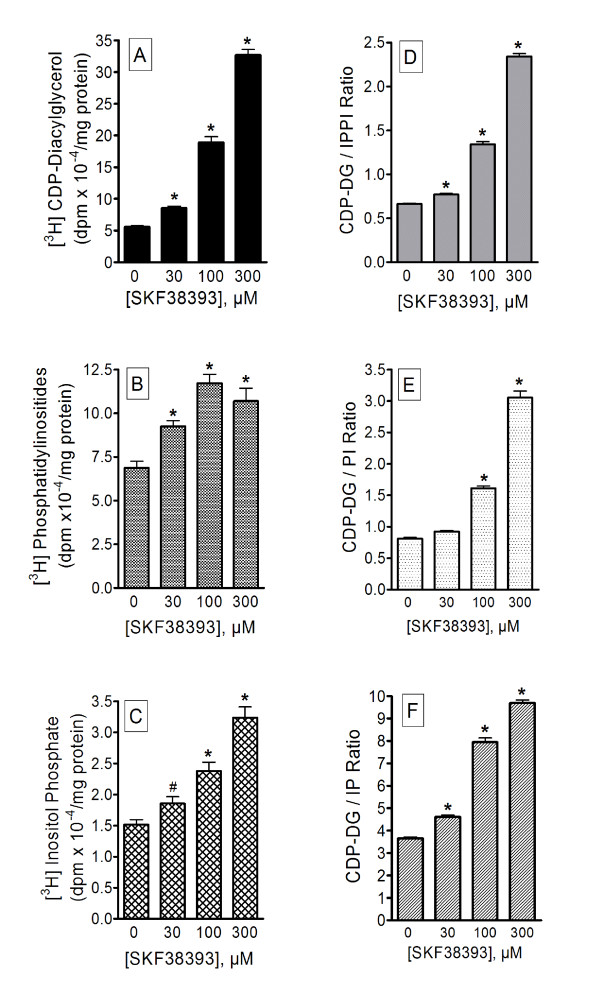
**Effects of SKF38393 on CDP-diacylglycerol production, inositol phosphate accumulation and phosphatidylinositide synthesis**. Prefrontal cortical slices pre-labeled with [^3^H]cytidine or [^3^H]inositol in parallel were incubated with indicated concentrations of SKF38393 in the presence of 5 mM LiCl for 90 min, followed by assay of [^3^H]CDP-diacylglycerol (**A**), [^3^H]phosphoinositides (**B**) and [^3^H]inositol phosphates (**C**). Calculated ratios of CDP-diacylglycerol over inositol phosphates (CDP-DG/IP ratio), phosphatidylinositides (CDP-DG/PI ratio), or the sum of the inositol phosphates and phosphatidylinositides (CDP-DG/IPPI ratio) are shown in the right panel (**D-E**, respectively). Each bar is the mean ± SEM (*n *= 12). Data were analyzed by One-way ANOVA and *posthoc *Dunnett tests. #p < 0.05; *p < 0.01, compared to the respective control by Dunnett test.

In attempts to determine which, if any, of the endogenous monoaminergic systems may show similar profiles of CDP-DG/IP effects, additional experiments were conducted with agonists that act directly at PLC-coupled monoaminergic receptors: α-methylserotonin (5HT_2 _serotonergic), carbachol (muscarinic cholinergic), SKF38393 (D_1_-like dopaminergic), and phenylephrine (alpha-adrenergic). Corresponding CDP-DG ratios were calculated as for the antidepressant agents. As shown, α-methylserotonin, carbachol, phenylephrine (Figure 9) or SKF38393 (Figure 10) significantly increased IP accumulation and CDP-DG production in frontal cortex or hippocampal tissues. Carbachol failed to increase PI synthesis, SKF38393 significantly enhanced PI synthesis, while the other two agents had significant but relatively small effects on PI. The ratios of CDP-DG production relative to IP accumulation are shown in the corresponding right panels of Figure 9 and Figure 10. With both carbachol and phenylephrine, there was a dramatic decrease in the CDP-DG/IP ratio. While the ratio did not decrease as much for α-methylserotonin, there was no concentration-related increase either. Conversely, SKF38393 increased CDP-DG/IP ratios significantly and in a manner similar to the antidepressants (Figure 10). Indeed, even the ratios of CDP-DG relative to PIs or the combination of both inositol derivatives (CDP-DG/IP&PI) were significantly enhanced. Thus, agonists at the direct PLC-coupled monoamine receptors showed parallel and corresponding changes between CDP-DG and the inositides, except for the dopamine agonist which, like the antidepressants, induced proportionately greater production of CDP-DG relative to inositide derivatives.

## Discussion

Various experimental approaches have been used in the past to demonstrate positive effects of select antidepressant agents on the IP arm of PI signaling cascades [15,27,30,66]. The present data demonstrate for the first time that antidepressant agents could increase by several-fold the production of CDP-diacylglycerol, a crucial signaling intermediate that is both a derivative of diacylglycerol and a precursor for the biosynthesis of PIs. This effect appeared to be common across agents from diverse chemical and pharmacological classes, seeing it was obtained with the tricyclics imipramine and desipramine, the SSRIs fluoxetine and paroxetine, the atypicals maprotiline and nomifensine, and the MAO inhibitors phenelzine and hydralazine. Thus, the findings could point to a mechanism (enhanced phospholipid biosynthesis) and mediator (CDP-diacylglycerol) for the biochemical and possibly clinical effects that may be common across diverse classes of antidepressants.

Earlier studies observed that several antidepressants enhanced [^3^H]IP accumulation and [^3^H]PI labeling in rat cortical slices [27,29]. The mechanism of this response was confounding, seeing other studies that directly assayed phospholipase C activity suggested that the drugs could stimulate or inhibit PLC-mediated PI hydrolysis [15,24]. In the present study, the antidepressants were equally effective in enhancing CDP-DG in the presence or absence of LiCl, whereas the presence of Li+ was necessary to demonstrate the effects of the drugs on IP accumulation. This implies that the compounds do not inhibit IP breakdown (otherwise they would have substituted for Li+), and that their effects on IP accumulation is probably secondary and passive to the enhanced production of upstream CDP-DG and PI substrates.

Numerous agents acting at diverse receptor systems can enhance PI metabolism, but few such pure receptor agonists are known to exhibit an antidepressive effect in humans or animals [67]. How then might an effect of antidepressant agents on CDP-DG and PI synthesis be associated with the antidepressive efficacy of the compounds? An attempt to address this question led to comparisons of the ratio data between the antidepressants as a group and agonists at alpha-adrenergic, 5HT_2 _serotonergic, and dopaminergic receptors (which are implicated in depression) as well as the muscarinic cholinergic receptor that is not known to contribute to the actions of antidepressant agents. While all these receptors are coupled to PI hydrolysis, only some 5HT_2 _agonists and SKF38393 have been shown to elicit antidepressive effects in rodent models [67-69]. The ratio of CDP-DG to IP components for phenylephrine and carbachol decreased, for α-methylserotonin remained unchanged, and for SKF38393 increased, with increasing concentrations of agonist. For the antidepressant agents, the ratios were not only significantly elevated, but actually increased in a concentration-dependent fashion. This was true of all classes of antidepressants examined, including the effective members among the MAO inhibitors. The similarity between the effects of SKF38393 and the antidepressants may underlie the behavioral antidepressant efficacy of the compound as previously demonstrated in the rodent model [69]. Hence, to the extent that CDP-DG production might be relevant to depression or the mechanism of antidepressant drug action, an antidepressive agent should not merely increase PI hydrolysis, but the CDP-DG produced must exceed and probably precede the production of PI messengers.

It was noteworthy that even the SSRIs produced significant increases in each CDP-DG ratio, whereas the direct 5HT_2 _agonist α-methylserotonin did not. If the biological actions of the SSRIs were limited to the actions of the drugs to enhance synaptic serotonin levels, then one would expect direct serotonin receptor stimulation to elicit similar effects. This disparity should suggest that facilitation of synaptic serotonin levels may not be the sole or sufficient mechanism of action of the SSRIs. Rather, antidepressant-enhanced synaptic serotonin may work in concert with antidepressant-facilitated neurolipid biosynthesis to achieve the type and level of downstream signaling responses that may contribute to the antidepressive effect.

Although CDP-DG production induced by the antidepressants may be derived from phosphoinositide breakdown, it is not impossible that the CDP-DG pool may be generated from additional endogenous sources. In an initial attempt to address this, we observed that antidepressant-mediated accumulation of [^3^H]CDP-DG was completely blocked by the non-specific phosphoinositide inhibitor, neomycin, in prefrontal or hippocampal tissues. Conversely, the selective PLC inhibitor U73122 could only partially decrease CDP-DG production, while it completely blocked the release of IPs. These results suggest that, while the integrity of the phosphoinositide pool is essential to the full effect of antidepressant agents on CDP-DG (based on the neomycin data), the PLC-accessible pool of phosphoinositides may not be the only source of antidepressant-mediated CDP-DG production (based on the U73122 data). It is known that neural (and other) cells maintain multiple pools of phosphoinositides not all of which may be accessible to PLC-mediated cycling. The possibility that the antidepressants could mobilize these additional reserves of PI substrates, particularly following acute or chronic metabolic depletion of the substrates, should be an interesting subject for future investigations.

A critical question that was also attempted relates to the extent to which the CDP-DG response may be specific to antidepressant agents versus other psychotropic drugs. After testing a wide range of compounds, we observed that neither the antipsychotics chlorpromazine and haloperidol, nor several other psychotropic agents were capable of inducing the degree of CDP-DG effects observed with the antidepressant agents. While this suggests that the CDP-DG effect, particularly the dose-related effect on CDP-DG/IP ratio, could reflect a characteristic property of antidepressant medications, we were equally surprised by the disparity in efficacy among the MAO inhibitors. The ineffective agents included the nonselective MAO A/B inhibitor clorgyline, and the selective MAO-B inhibitors pargyline and selegiline. At least one of these, clorgyline, is used clinically for the treatment of depression. Conversely, other MAO inhibitors, including phenelzine, hydralazine and tranylcypromine were significantly effective in inducing CDP-DG. The chemical or biological basis for this disparity among the MAOIs is still unclear. Indeed, considering the relatively marked effects of phenelzine and hydralazine, it is possible that inhibition of MAO-mediated monoamine breakdown may not be the predominant mechanism by which these compounds modulate CDP-DG signaling.

It remains to be determined how the present *in vitro *observations may relate to *in vivo *drug concentrations or behavioral effects. While all tested antidepressants generally induced significant CDP-DG or PI effects at concentrations of 1–10 μM (and as low as 0.1–0.3 μM for phenelzine and hydralazine), the in vivo concentrations or doses needed to induce comparable effects have not been determined. Nevertheless, a recent report suggests that antidepressants may induce *in vivo *CDP-DG or phosphoinositide effects at doses commonly used to elicit antidepressant-like behaviors in animal models [70]. Additional studies should help to clarify these questions.

## Conclusion

Collectively, the present data raise the speculation that depression may be associated with decreased turnover or biological efficacy of phosphoinositide-related signaling systems, possibly due to depletion of phosphoinositide substrates. Antidepressants may act by mobilizing CDP-DG to help replenish or supplement the pool of available PIs. In addition to PLC-mediated cascades, phosphatidylinositol is a key substrate for the PI3K signaling pathway [71]. Earlier reports showed that PI3K signaling is relevant to the induction of neurogenesis or neuronal survival and plasticity [72,73]. A recent study determined that the activity of PI3K and its downstream target, Akt, is decreased in postmortem brain tissues of depressed suicide victims [74]. Extensive interactions or crosstalk exists among downstream mediators of the PLC and PI3K systems and other signaling pathways that have been implicated in depression. Thus, it is conceivable that an early action of antidepressant agents to enhance the mobilization of CDP-DG could lead to coordinate effects on multiple signaling systems, which might then explain the various molecular, structural, and functional effects of the drugs. The data, however, do not exclude the possibility that multiple signaling pathways, including the adenylyl cyclase pathway, may be involved in depression or the mechanism of action of antidepressant agents. Nor do the data directly address the conventional notion that antidepressive medications need be administered chronically in order to elicit a clinical effect. Additional studies also are required before a model could emerge that might relate these findings to the various theories of depression or antidepressant mechanisms. Notwithstanding these pending questions, the present CDP-DG findings demonstrate a functional biochemical response that is common across divergent antidepressant classes, is elicited within the time frame that the drug is available to the tissues, and may have a plausible role in the pathology of depression or the mechanism of action of current antidepressive agents.

## Methods

### Animals

Male Sprague-Dawley rats weighing 225–250 g were obtained from Zivic Laboratories (Zelienople, PA) and housed in climate-controlled facilities with a 12-h light/dark cycle for at least 3 days before use. The animals were caged in groups of three and allowed free access to food and water. Protocols for the care and use of the experimental animals were approved by the Institutional Animal Care and Use Committee and conformed to the National Institutes of Health Guide for the Care and Use of Laboratory Animals.

### Drugs and chemicals

Antidepressant compounds and buffer reagents were purchased from Sigma-Aldrich (St. Louis, MO). SKF38393 was a gift from the NIMH Chemical Synthesis Program (NIMH, Bethesda, US). Nomifensine was first dissolved in 0.2% tartaric acid and SKF38393 in distilled water before either drug was diluted to use concentrations in assay buffer. Other drugs were prepared fresh in HEPES bicarbonate assay buffer (HBB) [65]. Each experiment was performed on multiple occasions using fresh preparations of drugs. Protein was assayed by the Bradford method using BioRad protein assay reagents (BioRad, Hercules, CA).

### Measurement of CDP-diacylglycerol accumulation

Accumulation of CDP-diacylglycerol was measured in brain slice preparations by taking advantage of the CTP-phosphatidate transfer reaction as previously described in detail [75-77]. Briefly, male Sprague-Dawley rats weighing between 225 and 300 g were rapidly decapitated and the brains removed and rinsed in calcium-free HBB [75,78]. Brain regions of interest, including the prefrontal cortex, hippocampus, and striatum, were quickly dissected out and 350 μm prisms prepared using a McIlwain tissue chopper [78]. The slices were washed with calcium-free HBB and pre-incubated for 45 minutes at 37°C. Slice aliquots of approximately 300 μg protein were then incubated with 1.5 μCi of 5- [^3^H]cytidine (20 Ci/mmol; American Radiolabeled Chemicals, St. Louis, MO) in order to generate an endogenous pool of radiolabeled cytidine triphosphate (CTP) for feeding into the CTP:phosphatidate transfer reaction [75]. Following addition of 5 mM LiCl, test drugs or buffer were added for a total volume of 250 μl, and incubation continued for 60 or 90 min as indicated. Reactions were terminated by addition of 1.5 ml chloroform-methanol-1M HCl (100:200:1). Formed lipids were extracted by liquid partitioning in chloroform followed by centrifugation at 1000 × g for 5 min in order to separate the liquid phases. Aliquots of the organic phase were quantitatively transferred into scintillation vials, dried at room temperature, and redissolved in Biosafe scintillation cocktail. Radioactivity in this lipid fraction was determined by liquid scintillation spectrometry; this activity corresponds to [^3^H]CDP-DG as previously indicated [63,75,79], and confirmed by us through thin layer chromatographic analysis of the reaction products (unpublished observations).

### Measurement of inositol phospholipid resynthesis

Brain tissues were prepared and incubated as described above for assaying CDP-DG, except that 1.5 μCi of [^3^H]inositol (20 Ci/mmol; American Radiolabeled Chemicals, St. Louis, MO) was used instead of [^3^H]cytidine to label the slices. Following the labeling incubation, drugs were added and allowed to act for 60 or 90 min as indicated. Samples were extracted with chloroform-methanol-1M HCl (100:200:1), partitioned with chloroform into aqueous and organic phases, and aliquots of the organic phase dried and assessed for radioactivity that corresponded to the inositol phospholipids. For purposes of the present study, it was not necessary to attempt to separate the multiple phosphorylated or isomeric forms of these phospholipids. Hence, the data potentially represent the mix of phosphatidylinositol, phosphatidylinositol-4-phosphate, and phosphatidylinositol 4,5-bisphosphate in any of their positional isomeric forms. Based on the levels of the phospholipids present at the start of drug treatment, a subsequent decrease is seen as depletion, whereas an increase in the [^3^H]inositol-labeled pool of the phospholipids is considered to represent further phospholipid synthesis or resynthesis [76,80].

### Measurement of inositol phosphate accumulation

To measure the levels of IPs formed, tissues were treated exactly as in the foregoing PI synthesis assays, including the use of [^3^H]inositol for prelabeling of the PI pool. The 250 μl reactions were terminated by mixing the samples with 1.5 ml of chloroform – methanol – 1 M HCl (100:200:1). Following chloroform-mediated partitioning of the extracts as described [65], aliquots of the aqueous phase were analyzed for the content of [^3^H]IPs by Dowex anion exchange chromatography [65,78]. An IP fraction was collected from the eluate and the solution converted into a gel by use of Scintisafe Gel (Fisher Scientific, Pittsburgh, PA). The amounts of IP-associated radioactivity in the samples were then measured by liquid scintillation spectrometry.

### Data analysis

Data from the various experiments were normalized relative to the respective control or basal measurements, and then pooled for analysis. Data were tested by an appropriate analysis of variance (ANOVA) using SPSS software (SPSS, Chicago, IL, USA). Where warranted, the ANOVAs were followed by *post hoc *analyses using the Dunnett test to compare various treatment means to their respective controls. Statistical comparisons were considered significant at *p *< 0.05 or better.

## Authors' contributions

KRT participated in experimental design, carried out the majority of the experiments, and drafted the manuscript. HOUV participated in data collection. ASU conceived of the study, directed the experiments, and participated in the data analysis and writing of the manuscript. All authors read and approved the final manuscript.
